# Evaluating Determinants of Length of Stay in Burn Care: Is One Day per 1% Total Burn Surface Area Still Accurate?

**DOI:** 10.7759/cureus.77473

**Published:** 2025-01-15

**Authors:** Armein Rahimpour, Nathan Fox, Errington C Thompson, Semeret Munie, Curtis W Harrison, David Denning, Paul Bown, Rahman Barry

**Affiliations:** 1 General Surgery, Marshall University Joan C. Edwards School of Medicine, Huntington, USA; 2 Trauma and Surgical Critical Care, Marshall University Joan C. Edwards School of Medicine, Huntington, USA; 3 Bariatric Surgery, Marshall University Joan C. Edwards School of Medicine, Huntington, USA; 4 Plastic and Reconstructive Surgery, Marshall University Joan C. Edwards School of Medicine, Huntington, USA

**Keywords:** appalachia, burn care, burn intensive care unit, inhalational injury, resource allocation, total hospital duration

## Abstract

Background

Despite advances in burn care, morbidity and mortality remain high. There is a large gap in research focusing on resource-limited Appalachian burn victims. Appalachia is unique in many different ways. The region is well known nationally for poor healthcare outcomes, household income below the national average, ranking high in addiction and drug use crisis, and characterized with a high prevalence of comorbidities such as chronic obstructive pulmonary disease (COPD), diabetes mellitus (DM), coronary artery disease, and obesity. To complicate this devastating imbalance, Cabell Huntington Hospital is the sole burn intensive care unit in the state of West Virginia, with only six beds available. It is crucial to understand the factors that prolong the length of stay (LOS), as LOS is a key indicator for healthcare resource utilization, especially in this resource-limited population. This study aims to identify factors that influence LOS among burn patients in Appalachia, focusing on demographic and clinical variables.

Methodology

A retrospective analysis was conducted among 748 patients between January 1, 2017, and January 1, 2023. Demographic and clinical variables, including age, gender, COPD, DM, smoking history, inhalational injury, burn source, body mass index (BMI), total burn surface area (TBSA), and total ventilation duration (TVD), were collected. Multiple linear regression was used to identify predictors of LOS. Statistical significance was set at p-values <0.05.

Results

Significant predictors of prolonged LOS included TVD (β = 1.25, p < 0.001), TBSA (β = 0.60, p < 0.001), inhalational injury (β = 6.02, p < 0.001), and burn source (thermal contact with metal: β = 10.68, p = 0.003). Discharge status (dead) was associated with shorter LOS (β = −17.09, p < 0.001). For every additional day of ventilation, LOS increased by approximately 1.25 days. Each percentage increase in TBSA contributed to a 0.6-day increase in LOS. Patients who died had a hospital stay approximately 17 days shorter than those who survived. The presence of inhalational injury extended the LOS by an average of six days. Age, gender, COPD, DM, BMI, and smoking history were not significantly associated with LOS.

Conclusions

Newer predictor models should be used to combine TBSA with other demographics, comorbidities, and burn factors, such as inhalation injury and TVD, to provide a more accurate LOS for patients, their loved ones, and caregivers. The rule that for every 1% TBSA burned LOS increases with one day does not hold in our population. These findings provide valuable insights for optimizing burn care in resource-limited settings.

## Introduction

Despite advances in burn care, morbidity and mortality remain high [1]. At present, burns are considered the fourth most common cause of trauma worldwide [2]. Considerable resources are spent on this preventable traumatic injury, with each burn patient averaging 88,218 US$ in total healthcare costs [1,2]. The longer the hospital duration, the more resources will be used, and the greater the healthcare burden. There are vast amounts of research published on how to reduce hospital stays and improve burn care with multidisciplinary care including specialized burn intensive care units (BICUs) [3]. However, there is a large gap in research focusing on resource-limited Appalachian burn victims.

Appalachia is unique in many different ways. This region is well known nationally for poor healthcare outcomes [4]. The average household income is below the national average, ranks high in addiction and drug use crises, and is characterized by high rates of comorbidities such as chronic obstructive pulmonary disease (COPD), diabetes mellitus (DM), coronary artery disease, and obesity [5-8]. The Appalachian population is more likely to fall victim to this preventable injury as it is well-known that burn victims are usually from low to middle-income households [1]. West Virginia, in particular, ranks among states with worse health outcomes in the nation due to limited healthcare access, multiple undiagnosed comorbidities due to inadequate primary care follow-up, low physical activity, and poor dietary habits [7,9,10]. To complicate this devastating imbalance, Cabell Huntington Hospital (CHH) houses the sole BICU in West Virginia, with only six beds available. This BICU receives patients from the tristate region (West Virginia, Kentucky, Ohio). It is of crucial importance to understand which factors prolong the length of stay (LOS), as it is a key indicator for healthcare resource utilization, especially in this resource-limited population.

This study aims to identify the factors influencing LOS among burn patients in Appalachia, focusing on demographic and clinical variables such as age, gender, comorbidities, inhalational injury, and burn severity. The findings aim to guide resource allocation, optimize patient outcomes, and address the unique healthcare challenges in this underserved region.

## Materials and methods

The study was approved by the Institutional Review Board (approval number: 2063568-1) at Marshall University. Patient records were retrospectively reviewed from our registry at CHH’s BICU. This hospital is an academic teaching hospital, regional referral center, and American College of Surgeons-verified Level 2 Trauma Center in Huntington, West Virginia. The data spanned January 1, 2017, to January 1, 2023. The dataset was curated from the hospital’s registry and obtained through a formal request to the Information Technology (IT) department. The inclusion criteria targeted all patients presenting with burn injuries during the study period.

Data extraction included demographic variables (age, gender), clinical variables (history of COPD, home oxygen use, smoking history, DM), and burn-specific metrics (source of burn, inhalational injury, body mass index (BMI), total burn surface area (TBSA), and LOS). The presence of inhalational injury was defined by clinical findings of carbonaceous material or soot in the oropharynx accompanied by difficulty in oxygenation. TBSA was calculated using the Wallace rule of nines.

Patients without documented burn diagnoses, including cases of Stevens-Johnson syndrome, road rash, frostbite, or unrelated conditions, were excluded after manual chart review. The final dataset comprised 748 patients after excluding those with misdiagnosed or non-burn injuries.

Statistical analysis

Descriptive statistics summarized the cohort’s characteristics. Continuous variables were expressed as means ± standard deviations (SDs). Categorical variables were presented as frequencies (N) and percentages (%). The relationships between potential predictors and LOS were assessed using chi-square tests for associations between categorical variables and Pearson’s correlation for continuous variables. Multiple linear regression was performed to quantify the impact of age, gender, COPD, home oxygen use, smoking, DM, source of burn, BMI, inhalational injury, TBSA, and total ventilation duration (TVD) on LOS. Significance thresholds were set at p-values <0.05. Statistical analysis was conducted using Python’s statsmodelslibrary.

## Results

The study analyzed data from 748 patients admitted for burn injuries between January 1, 2017, and January 1, 2023. Descriptive statistics, correlation analyses, and multiple linear regression were performed to assess the relationship between patient characteristics and LOS.

Descriptive statistics

The cohort consisted of 748 patients. As shown in Table 1, the gender distribution included 408 (54%) males and 340 (46%) females. The prevalence of comorbidities included COPD at 32%, DM at 22%, and smoking history at 44%. Inhalational injury was identified in 15% of patients.

**Table 1:** Summary of variables and their association with length of stay. The chi-square (χ^2^) test was used, and p-values <0.05 were considered significant. DM: diabetes mellitus; COPD: chronic obstructive pulmonary disease

Variables	n (%)	P-value	χ^2^
DM	165 (22)	0.566	0.57
COPD	239 (32)	0.405	0.83
Home oxygen use	150 (20)	0.235	-1.19
Smoking history	329 (44)	0.05	1.97
Inhalation injury	112 (15)	<0.001	4.1
Gender		0.498	0.68
Male	404 (54)		
Female	344 (46)		

Correlation analysis

Pearson’s correlation coefficients were calculated for continuous variables. For TBSA, a strong positive correlation with LOS was seen (r = 0.61, p < 0.001). TVD also correlated positively with LOS (r = 0.58, p < 0.001). However, BMI and age exhibited weak correlations with LOS (r = 0.12, p = 0.041 and r = 0.09, p = 0.078, respectively).

Multiple linear regression

As shown in Table 2, the mean age of the cohort was 58.3 ± 12.4 years. Furthermore, the cohort’s mean BMI was 18.7 ± 3.2 kg/m^2^, mean TBSA was 12.6% ± 8.7%, and mean TVD was 9.5 ± 2.6 days. A multiple linear regression model was applied to evaluate the independent effects of clinical and demographic factors on LOS. Significant predictors were TVD, TBSA, discharge status, and inhalation injury. TVD showed a positive association with LOS (β = 1.2466, SE = 0.069, p < 0.001), and for every additional day of ventilation, LOS increased by approximately 1.25 days. TBSA also showed a positive association with LOS (β = 0.6007, SE = 0.046, p < 0.001), and each percentage increase in TBSA contributed to a 0.6-day increase in hospital stay. For patients who expired, a significant negative association with LOS was noted (β = −17.0917, SE = 2.821, p < 0.001). Patients who died had a hospital stay approximately 17 days shorter than those who survived. Inhalational injury also exhibited a positive association with LOS (β = 6.0164, SE = 1.467, p < 0.001). The presence of inhalational injury extended LOS by an average of six days. Finally, the source of burn (thermal, contact, metal) had a positive association with LOS (β = 10.6813, SE = 3.606, p = 0.003). Patients with this burn source had significantly longer hospital stays.

**Table 2:** Summary of variables and their association with length of stay. The t-test (t-score) was used, and p-values <0.05 were considered significant. SD: standard deviation

Variables	Mean ± SD	P-value	t-score
Age (years)	58.3 ± 12.4	0.096	1.67
Total ventilation duration (days)	9.5 ± 2.6	<0.001	17.96
Body mass index (kg/m^2^)	18.7 ± 3.2	0.224	-1.22
Total burn surface area (%)	12.6 ± 8.7	<0.001	13.1

The following factors were not significantly associated with LOS: age (β = 0.0599, p = 0.096), BMI (β = −0.0558, p = 0.224), gender (β = −0.6822, p = 0.498), COPD (β = 1.6912, p = 0.405), home oxygen use (β = −4.1358, p = 0.235), and cigarette smoking (β = 1.8544, p = 0.050).

## Discussion

The focus of this study was to understand the factors that influence LOS in the resource-limited Appalachian burn center. This study reviewed data from the sole BICU in the state of West Virginia over a six-year span.

The mean age of our study population was 58 years. This age is higher than the national average [11,12]. For example, in one national burn database study where patients younger than 18 years were excluded, the average age of burn patients was 43.5 years. This discrepancy can be explained by many factors unique to our Appalachian population. The state of West Virginia has the oldest population in the United States, with an average age higher than the national average [13]. In addition, as mentioned earlier, the majority of the population in West Virginia lives in rural areas, has lower socioeconomic status, and relies on unsafe home heating practices that are a common source of burn injuries.

Another unique study finding was that the burn victims in our population were equally distributed between genders, with 54% males and 46% females. Most national studies show males are more at risk for burn injuries [11,12]. The National Burn Repository from the American Burn Association (2023) noted that the median age of their population was 39 years, with over 65% being male [12]. Another national database from 2024 reported this figure to be 73% [11]. This discrepancy can partially be explained by the unique patient population we serve, where the rural setting allows females to be more involved in high-risk environments such as farming, food preparation in large-scale kitchens, or using open-flame heating methods. These practices often rely on older heating systems, kerosene stoves, or wood stoves, leading to more burn injuries.

Our patient population’s mean TBSA of 12.6% was similar to the national average of 11.5% [11]. The prevalence of comorbidities was higher in our population than the national average. For DM, the prevalence in our population was almost double the national average. The Centers for Disease Control and Prevention reported the national DM prevalence to be 11.3% compared to 22% in our population [14]. This can be attributed to poor dietary choices, lack of physical activity, and decreased healthcare access [9,15]. For COPD, our population had a 32% prevalence compared to the national average of 6.4% [15]. Smoking prevalence in our population was 44% compared to the national average of 14% [16]. Inhalation injury for our population was 15%. Variations in reported prevalence rates for inhalation injury range from 5% to 30% [17-19]. The definition of inhalation injury and its diagnosis often differs between studies which could explain this discrepancy.

Our study indicated that LOS was positively correlated with TBSA and TVD. Although a positive correlation was seen between BMI and LOS, this association was weak. Further analysis evaluated the independent effects of each variable on LOS. We observed that for every additional day of ventilation and every percentage increase in TBSA, LOS increased by approximately 1.25 days and 0.6 days, respectively. These findings are useful for improving patient-doctor discussions, setting realistic expectations, and helping BICU members anticipate the duration of LOS. A retrospective review from a national repository found that, in general, LOS is positively correlated with TBSA, and the one-day per 1% TBSA rule is a useful guideline but not always accurate [20-22]. Our study indicated that this relationship might be overestimated, with our findings suggesting 0.6 days per 1% TBSA. Further research should focus on stratifying TBSA and examining moderate-to-severe burns (>20% TBSA). Newer predictor models should integrate TBSA with other demographics, comorbidities, and burn-related factors, such as inhalation injury and TVD, to predict LOS more accurately for patients and their families. One study analyzed patients who did not follow the one-day per 1% rule [23]. Factors such as age, percentage of third-degree burns, inhalation injuries, and other comorbidities impacted the LOS [23]. One important factor that was pointed out was in-hospital complications such as bacteremia, pneumonia, sepsis, and similar complications [23]. Our study also confirmed that inhalation injury has a positive association with LOS, with inhalation injury extending LOS by an average of six days.

Discharge status impacted LOS for our population. Mortality had a negative association with LOS, as deceased patients had hospital stays approximately 17 days shorter in comparison to those who survived. One study showed that the timing of death followed a bimodal distribution indicating that hospital stays for deceased patients are not always shorter [24]. For patients who survived the resuscitation period, the majority of deaths occurred due to in-hospital complications such as sepsis, worsening chronic disease, and pneumonia [24].

Another factor that prolonged LOS was thermal contact burns with metal. Usually, these patients are diabetic and experience sensory loss in their extremities. Patients are often unaware of the contact, leading to deeper tissue damage, which, in turn, prolongs LOS due to further surgical management. Multiple factors that we anticipated to prolong LOS were non-significant, including age, gender, COPD, home oxygen use, and cigarette smoking.

​This study’s retrospective design may limit causal inferences, and its single-center design limits the generalizability of the findings. However, it is a unique study of West Virginia’s burn population. It will allow a more comprehensive understanding and LOS anticipation for caregivers, families, and loved ones.

## Conclusions

Many factors impact LOS for burn victims, particularly TBSA, TVD, and the presence of inhalation injury. Resource allocation to support the BICU at CHH is critical to address the unique healthcare challenges of Appalachia. These findings provide valuable insights for optimizing burn care in resource-limited settings and help clinicians better understand burn recovery and set realistic expectations for care.
